# Supplementary material for: Identification of photoperiod-induced specific miRNAs in the adrenal glands of Sunite sheep (Ovis aries)
Source: Front Vet Sci. 2022 Jul 22;9:888207. doi: 10.3389/fvets.2022.888207 (PMC9354845; doi:10.3389/fvets.2022.888207)
Supplement: Supplementary file 1 [file Data_Sheet_1.ZIP › Supplementary file/miRDeep2 file.zip!/miRDeep2 file/Know_Structure/SPLP42A2/oar-mir-3955.pdf]

miRBase precursor : oar-mir-3955
Total read count : 519
oar-miR-3955-5p read count : 470
oar-miR-3955-3p read count : 35
remaining reads : 14

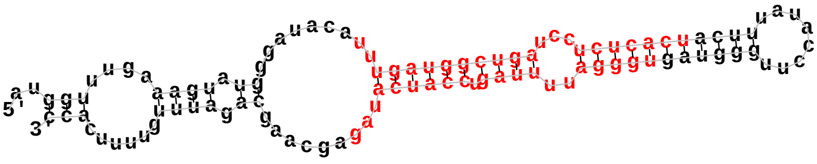

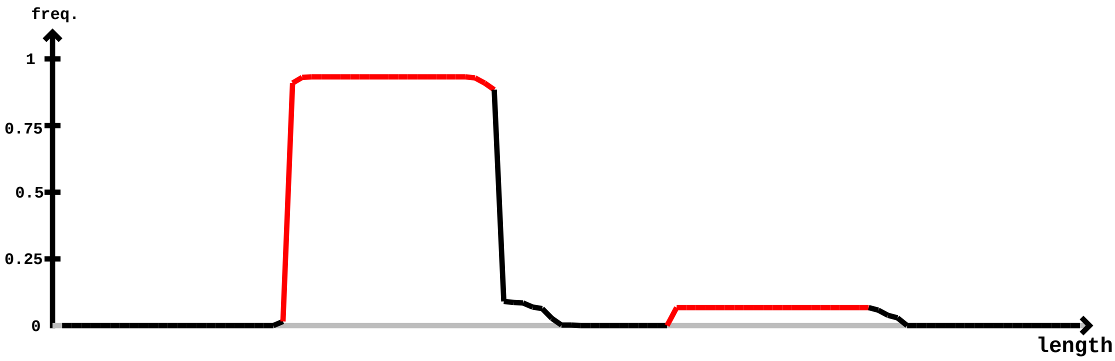


```
                                           oar-miR-3955-5p
                                                                              oar-miR-3955-3p
5'- augguuugaaaguaugggauacauuugauggcugauccucucacuacuuuauaccuuggguagugggauuuuaguccaucauagagcaagcagauuuguuuucacc  -3'   exp
    ..........((((...))))...((((((((((...((((((((((........))))))))))..)))).)))))).(((((((......)))))))....   reads   mm   sample
    ......................auuugauggcugauccucucacu.............................................................   8       0    seq
    .......................uuugauggcugauccucuc................................................................   2       0    seq
    .......................uuugauggcugauccucuca...............................................................   10      0    seq
    .......................uuugauggcugauccucucac..............................................................   13      0    seq
    .......................uuugauggcugauccucucacu.............................................................   394     0    seq
    .......................uuugauggcugauccucucacua............................................................   2       0    seq
    .......................uuugauggcugauccucucacuac...........................................................   1       0    seq
    .......................uuugauggcugauccucucacuacu..........................................................   6       0    seq
    .......................uuugauggcugauccucucacuacuu.........................................................   3       0    seq
    .......................uuugauggcugauccucucacuacuuu........................................................   19      0    seq
    .......................uuugauggcugauccucucacuacuuua.......................................................   13      0    seq
    .......................uuugauggcugauccucucacuacuuuaua.....................................................   1       0    seq
    ........................uugauggcugauccucucacu.............................................................   9       0    seq
    ........................uugauggcugauccucucacuacu..........................................................   2       0    seq
    .........................ugauggcugauccucucacu.............................................................   1       0    seq
    ...............................................................ugggauuuuaguccaucauag......................   5       0    seq
    ...............................................................ugggauuuuaguccaucauaga.....................   10      0    seq
    ...............................................................ugggauuuuaguccaucauagag....................   5       0    seq
    ...............................................................ugggauuuuaguccaucauagagc...................   15      0    seq
```
